# An Investigation into the Lifestyle, Health Habits and Risk Factors of Young Adults

**DOI:** 10.3390/ijerph120404380

**Published:** 2015-04-22

**Authors:** Yahya Al-Nakeeb, Mark Lyons, Lorna J. Dodd, Anwar Al-Nuaim

**Affiliations:** 1 College of Education, Qatar University, Doha 2713, Qatar; 2 Biomechanics Research Unit, Department of Physical Education & Sport Sciences, University of Limerick, Limerick, Ireland; E-Mail: mark.lyons@ul.ie; 3 Department of Psychology and Counselling, Newman University, Birmingham, B32 3NT, UK; E-Mail: l.dodd@newman.ac.uk; 4 Department of Physical Education, King Faisal University, Al-Ahsa, Saudi Arabia; E-Mail: NUAI200@newman.ac.uk

**Keywords:** clustering, risk factors, young adults, diet, physical activity, body mass index

## Abstract

This project examined the lifestyle, health habits and risk factors of young adults at Qatar University. It explored the clustering and differences in dietary habits, body mass index (BMI) and physical activity (PA) amongst male and female students, both Qatari and non-Qatari. Seven hundred thirty two students aged 18–25 years completed a self-reported questionnaire and an objective measure of BMI. Males and females had a high prevalence of being overweight and obesity and low levels of PA, according to well-established international standards. Three clusters were identified based on the students’ lifestyle and dietary habits. Cluster 1 (high risk factors) included those who engaged the least in healthy dietary practices and consumed the most unhealthy foods, participated in less PA and had the highest BMI. Cluster 2 (moderate risk factors) included those with considerably more habits falling into the moderate category, engagement in the most PA, the least TV and computer viewing time and had the lowest BMI. Cluster 3 (low risk factors) included those who engaged the most with the four healthy dietary practices, the least with the four unhealthy dietary practices and participated in moderate PA per week. This project provides valuable data that could be used by policy makers to address issues concerning student’s health.

## 1. Introduction

Physical inactivity and obesity are leading risk factors for global mortality [1]. The increase in the global obesity epidemic during the past few decades is substantial. However, there are wide variations in obesity prevalence across countries and populations due to socioeconomic, cultural and transport differences in national and local environments [2]. Industrial countries have witnessed significant technological advancement and automation during the first half of the 20th century. This was paralleled by decreases in food energy supply that helped in preserving low obesity prevalence. However, in the 1970s–1980s, an energy balance turning point seems to have occurred in many high-income countries [3], followed by a number of middle-income and low-income countries who have joined the global surge in obesity prevalence in adults and children [4,5].

It appears that the most obvious environmental precondition for a population to develop obesity is sufficient wealth and economic prosperity [5]. Since the discovery of oil in the Arabian Gulf region in the 1960s, the Gulf Cooperation Council (GCC) countries that comprise Bahrain, Kuwait, Qatar, Oman, Saudi Arabia and the United Arab Emirates (UAE) have experienced continued growth in population, per capita income and wealth. The UAE and Qatar in particular have grown the fastest in terms of population, per capita income and wealth [6]. With this growth, the Qatari population has witnessed significant lifestyle changes due to rapid urbanisation, the dominance of personal transport, the introduction of labour-saving devices, the availability of high-fat and dense-caloric foods, increased reliance on telecommunication technology, as well as decreased occupational-work demands [7,8]. These lifestyle changes have had a considerable impact on reducing the physical requirements of daily life and have encouraged sedentary lifestyles. This lifestyle transformation is thought to be greatly responsible for the significant increase in non-communicable diseases, such as cardiovascular disease (CVD), cancer and diabetes mellitus type II in Qatar [9]. Diabetes and CVD have become the leading causes of morbidity and mortality over the past two decades in Qatar [10].

The most important risk factors of non-communicable diseases in the Arabian Gulf countries include high blood pressure, high concentrations of cholesterol in the blood, inadequate intake of fruit and vegetables, being overweight or obesity, physical inactivity and tobacco use [9]. Five of these risks are closely related to inappropriate diet and physical inactivity. In the GCC countries, alarming levels of physical inactivity have been reported, as well as poor dietary practices, predisposing them to health problems [11,12,13]. To date, limited attempts have been made to examine the interrelationship of these risk factors within young adults. However, directional relationships have been identified in several studies. For example, previous research has demonstrated positive correlations between: (1) sugar-sweetened beverage consumption and poor dietary habits [14]; (2) skipping breakfast, lower nutritional status and increasing the risk of cardio-vascular disease [15]; and (3) low fruit and vegetable intake and low physical activity (PA) [16]. Consequently, whilst these studies have tended to focus on the significance of one unhealthy behaviour in isolation, research has shown that health behaviours often coexist with clear evidence of clustering [16,17,18,19,20,21,22,23,24,25,26].

Unhealthy lifestyle behaviours are modifiable and usually established during youth or young adulthood [27]. Furthermore, being overweight and obesity in youth are powerful indicators of being overweight in adulthood and related disease [28]. Despite the widely-documented consequences associated with unhealthy lifestyle behaviours, globally, a substantial proportion of young adults, notably university students, engage in unhealthy lifestyle practices [16,27]. The transition from school into university is normally coupled with a combination of stressors, which can have a significant impact on students’ health lifestyle choices [16]. This transitional period is critical for the development of lifelong healthy attitudes and practices, as well as for avoiding the biological precursors of chronic disease in later life. Of the limited number of studies examining the clustering of health lifestyle behaviours, very few have focused on university students. Consequently, the aims of this study were to: (1) explore the lifestyle, health habits and risk factors amongst young adults studying at Qatar University; (2) examine the interrelationships and clustering of risk factors, such as dietary habits, body mass index (BMI), smoking and PA; and (3) identify the reasons for participation in PA and the barriers for non-participation. The findings from this study should provide substantive information on PA, sedentary behaviour and the health habits of this important sector of the population who embody the nation’s future vitality. It should also enable health professionals to understand how behaviours cluster together, so that they can design more effective intervention strategies in the future.

## 2. Method

### 2.1. Participants

A representative sample of young adult males (*N* = 320) and females (*N* = 412) from Qatar University (QU) took part in this study following written informed consent along with institutional ethical approval. The sample represented the different colleges in QU, as well as the ratio of males to females; the sample size was determined so that it was within ±0.05 of the population proportion with a 95% confidence level. Incentives were provided to participants in order to encourage them to take part in the study.

### 2.2. Research Site and Procedures

This research was conducted within the Qatar University campus. Data collection and measurements were conducted at a number of specified areas that were convenient to reach by students from different colleges. Furthermore, private and screened testing spaces (one for males and one for females) were used for obtaining objective measurements of height and body mass. All measurements were taken by trained researchers of the same gender as the participants.

### 2.3. Lifestyle Questionnaire

A validated self-report questionnaire with 47 items was used to assess the PA patterns, sedentary activities (e.g., daily TV/computer/DVD viewing time) and dietary habits of the selected sample. For greater accuracy, the questionnaire was administered face-to-face by the researchers, thus enabling researchers to explain the questionnaire in full prior to completion. It also afforded participants the opportunity to pose any questions before, during or after completing the questionnaire. The questionnaire included the following two sections:

(1) PA section: This comprised a series of questions relating to various domains of PA representing light-, moderate- and vigorous-intensity physical activities. Each PA was assigned a metabolic equivalent (MET) value based on previous MET compendium guidelines for the PA of adults [29]. The average MET value assigned to moderate intensity sports was 4 METs, whilst vigorous intensity sports were assigned an average value of 8 METs. Slow (2.8 METs), normal (3.5 METs) and brisk (4.5 METs) walking pace was assigned modified values from the compendium of PA [30]. Based on the metabolic equivalent values of each activity, total energy expenditure per week was calculated (total MET-min per week), as well as energy expenditure per week spent in vigorous- and moderate-intensity PA. A cut-off point of 60 minutes per day of at least a moderate level of PA was used to classify overall activity level, based on recent recommendations [31]. The data were then converted into 3 activity categories based on total MET-min per week as follows: active, *>*1680 MET-min per week (60 min *×* 7 days *×* 4 METs); minimally active: *≥*840, *<*1680 MET-min per week (30 min *×* 7 days *×* 4 METs); inactive: *<*840 MET-min per week [32]. The PA questionnaire has been shown to have high reliability (intra-class correlation (ICC) = 0.85; 95% confidence interval level (CL) = 0.70 – 0.93) and acceptable validity (*r* = 0.30; *p* < 0.05) against pedometers [33].

(2) Dietary habits section: This included specific questions designed to quantify the frequency (servings per week) of certain dietary habits of youth. The questions covered healthy and unhealthy dietary habits, including how many servings per week the participants consumed for breakfast, fruit and vegetables, milk, sugary foods (donuts/cake), energy drinks, fried potatoes and fast foods. Both Western and Arabic fast food choices were included in the questionnaire.

### 2.4. Body Mass Index (BMI) Measurement

Body mass was measured using medical weighing scales (Seca Ltd., Hamburg, Germany) to the nearest 100 g. To ensure measurement accuracy, the scale was regularly calibrated in line with the manufacturer’s guidelines. The scales were also checked for a zero reading before each measurement. Height was measured using a portable height measure (Seca Ltd., Hamburg, Germany) to the nearest 0.5 cm. BMI was calculated using the formula: body mass (kg)/height (m^2^) in accordance with the World Health Organisation (WHO) criteria for overweight and obesity classification [34].

### 2.5. Statistical Analysis

Descriptive statistics were utilized to highlight the prevalence of being overweight/obesity, as well as classifications according to the activity index. PA levels, BMI, health and dietary habits of the young adults were analysed according to nationality and gender using two-way analyses of variance (ANOVA). Additionally, a two-step cluster analysis was used to identify clusters based on dietary practices, BMI, TV/computer viewing/playing and PA (e.g., total MET-min per week). This analysis identifies subgroups of cases in specific populations based on shared characteristics. Clusters are then given names based on the characteristics of the variables that shaped them [35,36]. The measurement criteria are the patterns within the cluster and how they differ from another cluster. Subsequent analyses, such as chi-square and analysis of variance (ANOVA), were used to identify differences between the clusters with regard to demographic characteristics (*i.e*., gender, age, nationality). Analyses were conducted using the Statistical Package for Social Sciences (Version 22, SPSS Inc. Chicago, IL, USA).

## 3. Results

**Figure 1:**
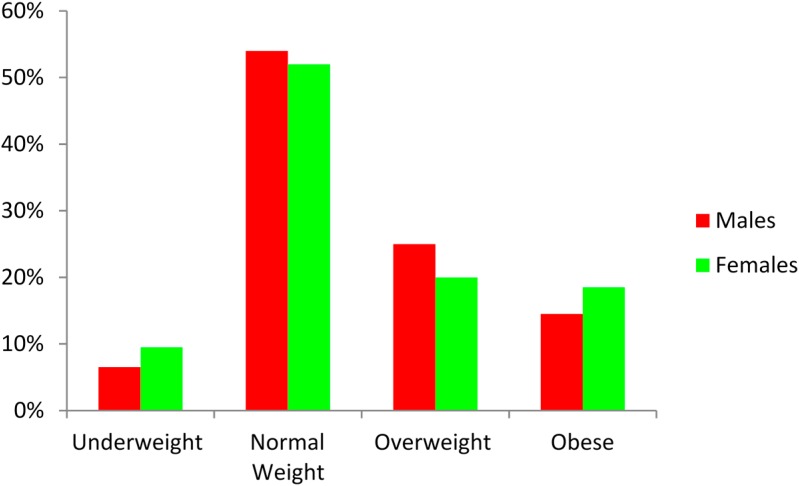
Body mass index (BMI) classification in males and females.

Table 1 presents the anthropometric characteristics of the study population. It is clear that 39.5% of males and 38.5% of females were overweight or obese. Figure 1 illustrates the percentage of males and females who were underweight, normal weight, overweight and obese according to World Health Organization (WHO) (2004).

**Table 1:** Anthropometric characteristics (mean ± SD) of the overall sample.

Gender	Age	Height	Weight	BMI
Males	22.0 ± 4.5	174.7 ± 6.9	76.2 ± 16.8	24.9 ± 5.1
Females	20.6 ± 3.1	161.9 ± 8.3	64.9 ± 17.9	20.6 ± 3.1

The results of this study revealed an increasing trend of being overweight and obesity among students across study Years 1 to 4. With regard to females, the percentage of overweight/obese students in Year 1 was 32%, and this had risen to 46% in Year 4. As for males, there were 36% overweight/obese in Year 1 and 55% in Year 4. Additionally, the percentage of students who are inactive appears to rise incrementally from 38.6% in Year 1 to 62.5% in Year 4 (Table 2). Meanwhile, the percentage of students who are active drops from 42.1% in Year 1 to 20.8% in Year 4.

Results also indicate that there is a notable difference in the level of physical activity of Qatari students and their non-Qatari counterparts. The percentage of inactive Qatari students is higher compared to non-Qatari students. Furthermore, the percentage of active students is higher among the non-Qatari students compared to the Qatari students. The trends here are consistent in both male and female students (Table 3).

**Figure 2:**
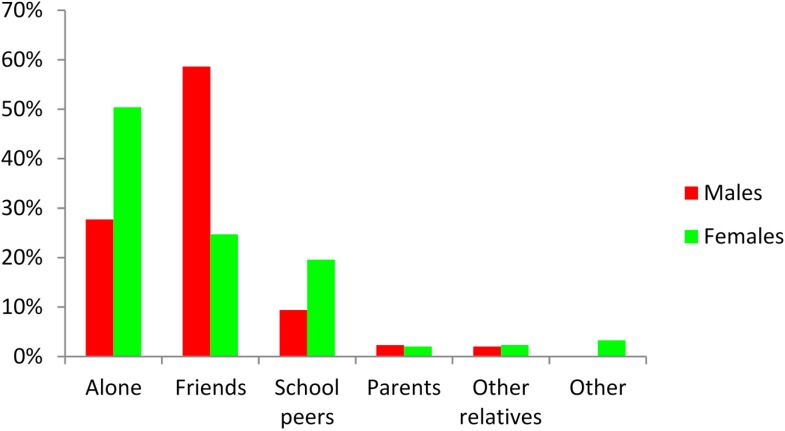
Responses of male and female students as to whom they engage with in physical activities or sport.

The activities/sports most regularly practiced by males were football 27.5%, gym 23% and walking 12.5%. Amongst female students, the activities/sports most regularly practiced were gym 20.5%, walking 18.5%, dance 17% and aerobics 11%. The majority of males (58.6%) stated that they engaged in activities or sports with their friends, whilst the majority of females (50.4%) engaged in activities alone. Figure 2 shows that there are marked differences in the responses of males and females with regard to whom they engage with in physical activities or sports.

**Table 2:** Levels of physical activity across study Years 1–4.

Year	Gender	Physical Activity Index (MET-min Per Week)		
		Inactive	Minimally Active	Active
**Year 1**	Males	17.7%	12.5%	69.8%
	Females	50.9%	23.3%	25.8%
	Total	38.6%	19.3%	42.1%
**Year 2**	Males	27.3%	12.2%	51.5%
	Females	52.5%	20.6%	27%
	Total	40.3%	20.9%	38.8%
**Year 3**	Males	28.9%	20%	51.1%
	Females	78.9%	5.8%	15.4%
	Total	55.7%	12.4%	32%
**Year 4**	Males	46.2%	19.2%	34.6%
	Females	71.7%	15.2%	13%
	Total	62.5%	16.7%	20.8%

**Table 3:** Levels of physical activity in Qatari and non-Qatari males and females.

Nationality	Gender	Physical Activity Index (MET-min Per Week)		
		Inactive	Minimally Active	Active
Qatari	Males	28.9%	20.3%	50.8%
	Females	61.8%	19.3%	18.9%
	Total	50.8%	19.6%	29.6%
Non-Qatari	Males	26.3%	16%	57.7%
	Females	49.1%	22%	28.9%
	Total	36.5%	18.7%	44.8%

**Figure 3:**
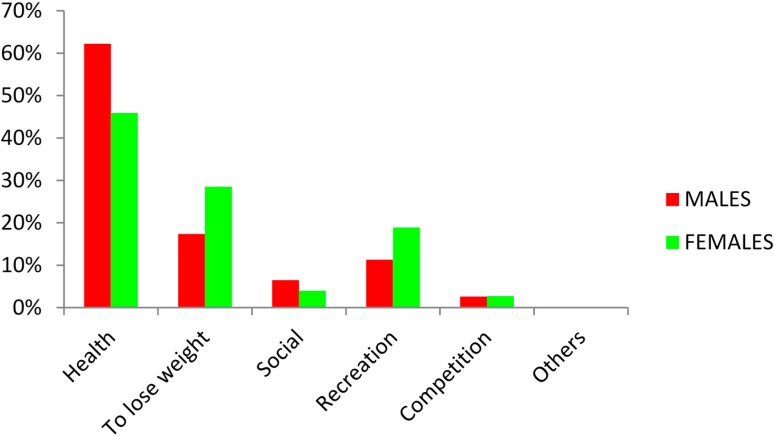
Reasons for regular participation in physical activities or sports.

Figure 3 outlines the main reasons for regular participation in physical activities or sports. The results indicated that health was the main reason for participation among both males (62.2%) and females (45.9%). Losing weight was cited by 28.5% of females as a key reason for regular participation in physical activities or sports compared to only 17.4% of males. The other reasons for participation in PA or sport are illustrated in Figure 3.

**Figure 4:**
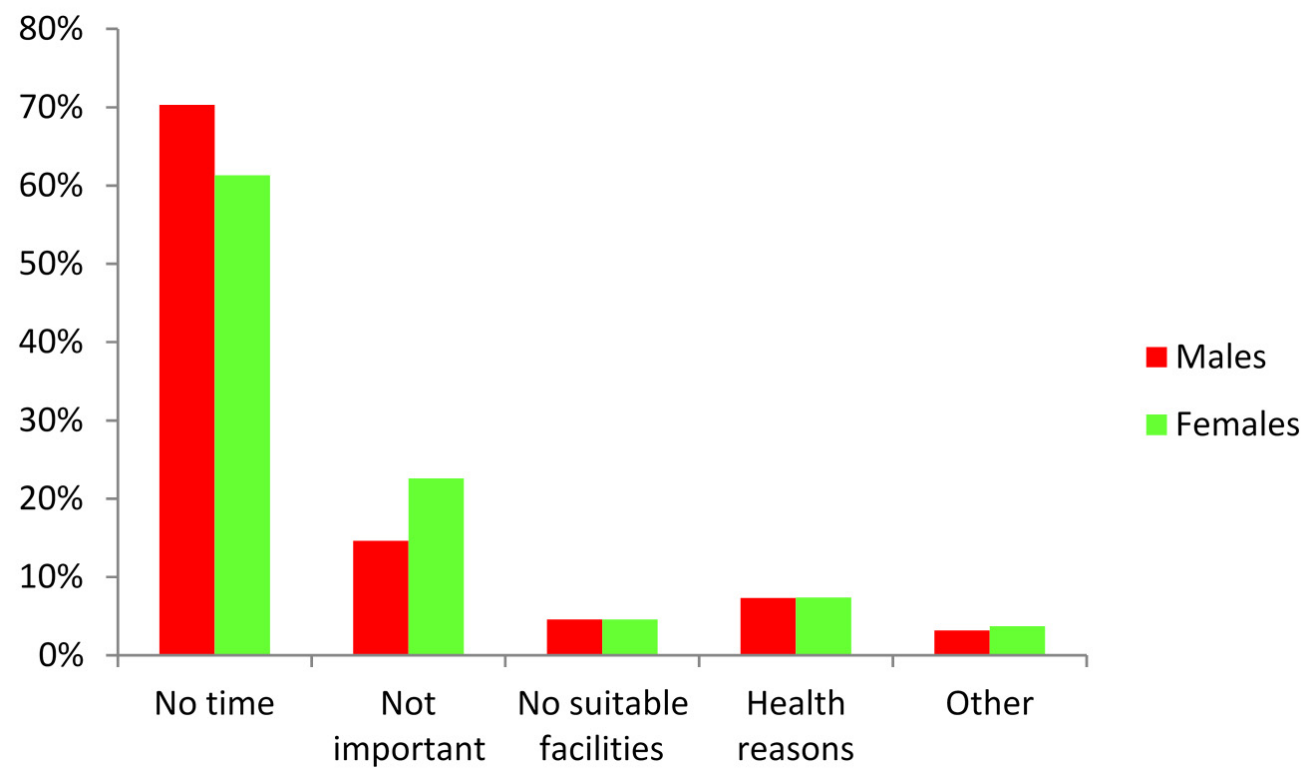
Main barriers to regular participation in physical activity or sport.

The barriers to regular participation in PA or sport were also explored. The findings (Figure 4) revealed that 70.3% of males and 61.3% of females reported a lack of time as the main reason for non-participation in PA. Participants also indicated that taking part in PA/sport was not important (14.6% and 22.6% of males and females, respectively).

**Figure 5:**
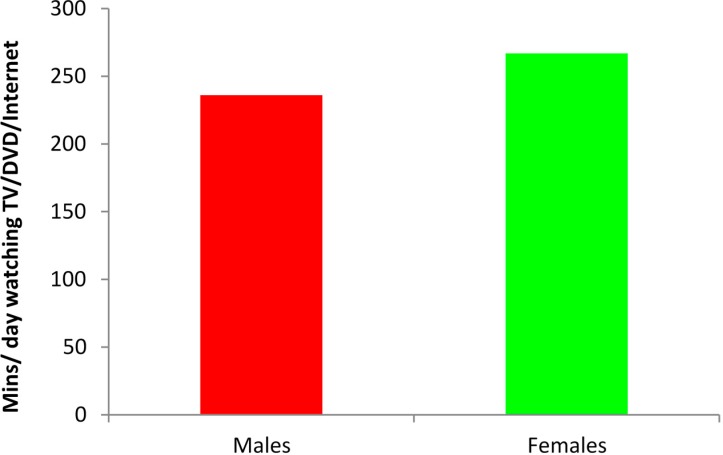
Combined time per day spent on computer/Internet, TV/DVD/video viewing.

Both male and female students reported lack of time as the main barrier to participation in PA or sport. However, Figure 5 illustrates clearly that males spent 3 h 56 min and females 4 h 26 min per day watching TV/DVD/video games/Internet use. In both cases, this is a significant proportion of waking hours per day.

The two-step cluster analysis identified three distinct clusters based on a number of risk factors (Table 4). Consequently, cluster names were based on the characteristics of the variables that shaped them. Cluster 1 (high risk factors) included those who engaged the least with healthy dietary practices and consumed the most unhealthy foods compared to the other two clusters. This cluster included those who participated in the lowest level of PA and had the highest BMI. Sedentary behaviour, such as TV viewing, was also greatest in this cluster. This cluster consisted mainly of females (70.6%) of Qatari nationality (51.2%), with a mean age of 20.4 years. Cluster 2 (moderate risk factors) included those who had considerably more habits falling into the moderate category compared to Clusters 1 and 3. This cluster was shaped by moderate fruit and vegetable intake per week, moderate breakfast consumption (on average, three times per week) and moderate consumption of milk. This cluster was also shaped by moderate sugary food intake, fast foods, fried potatoes and consumption of two energy drinks per week on average. Nevertheless, this cluster included those who reported high PA engagement (MET-min/week), low TV and computer game viewing/playing and low BMI. This cluster contained more males (69%) of non-Qatari nationality (56.3%), with a mean age of 20.9 years. Cluster 3 (low risk factors) included those who engaged the most with the four healthy dietary practices and the least with the four unhealthy dietary practices compared to Clusters 1 and 2 (Table 4). This cluster included those who participated in moderate PA/week and had a mean BMI of 24.9. This BMI is greater than that in Cluster 2, but lower than the BMI in Cluster 1. Similarly, TV viewing was greater in Cluster 3 than in Cluster 2, but less than in Cluster 1. Computer viewing was slightly greater in the participants in this cluster compared to Clusters 1 and 2. This cluster contained more females (58.7%) of non-Qatari nationality (56.4%), with a mean age of 22.4 years.

The pattern of cluster membership differed across gender (χ^2^_(2)_ = 51.633, *p* = 0.000, Cramer’s *phi* = 0.316) and mean age (F_(2,510)_ = 13.523, *p* = 0.000, eta squared = 0.05), but there was no significant difference (*p* > 0.05) between the three clusters with regard to Qatari and non-Qatari students. Further partitioning of the cluster * gender significant contingency table depicted the following; a significant association between Clusters 1 and 2 and gender (χ^2^_(1)_ = 51.075, *p* = 0.000, *phi* = −0.385), with a higher percentage of females (79.8%) in Cluster 1 and a higher percentage of males (57.6%) in Cluster 2. A significant association was found between Clusters 1 and 3 and gender (χ^2^_(1)_ = 6.037, *p* = 0.014, *phi* = 0.124), with a higher percentage of females (60.4%) in Cluster 1 and a higher percentage of males (52.6%) in Cluster 3. Finally, there was a significant association between Clusters 2 and 3 and gender (χ^2^_(1)_ = 22.513, *p* = 0.000, *phi* = 0.275), with a higher percentage of males (55.1%) in Cluster 2 and a higher percentage of females (72.1%) in Cluster 3. Significant findings were associated with small effect sizes. With respect to cluster * age, Tukey’s HSD *post hoc* tests revealed that there were highly significant (*p* < 0.001) differences between two of the clusters (1 * 3 and 2 * 3). Whilst older participants fell into Cluster 3 (mean: 22.4 years), the youngest participants fell into Cluster 1 (mean: 20.4 years).

**Table 4:** Descriptive statistics for the three clusters of risk factor behaviours.

Cluster	Cluster 1 (*n* = 218, 42.2%)	Cluster 2 (*n* = 126, 24.4%)	Cluster 3 (*n* = 172, 33.3%)
	High Risk Factors	Moderate Risk Factors	Low Risk Factors
Risk Factor behaviours	Mean	Mean	Mean
**Healthy Dietary Habits**			
Fruits (servings/week)	2.98	3.90	4.42
Vegetables (servings/week)	3.72	4.24	5.41
Breakfast (servings/week)	2.77	3.33	4.52
Milk (servings/week)	4.21	4.38	4.90
**Unhealthy Dietary Habits**			
Surgery foods (servings/week)	4.94	3.37	2.68
Fast foods (servings/week)	3.92	2.90	1.20
Fried potatoes (servings/week)	3.61	2.61	1.09
Energy drinks (servings/week)	0.40	2.02	0.10
**Physical Activity Behaviour**			
PA behaviour			
(total MET-min/week)	1241.79	4604.56	1351.06
TV viewing time (h/day)	3.26	2.14	2.80
Computer viewing time (h/day)	1.91	1.50	1.95
BMI (kg/m^2^)	25.15	23.43	24.90
**Demographic Factors**	**%**	**%**	**%**
**Gender**			Cramer’s *phi* = 0.316 ^***^
Male	29.4	69.0	41.3
Female	70.6	31.0	58.7
			Eta Squared = 0.05 ^**^
**Age**	20.4	20.9	22.4
**Nationality**			Cramer’s *phi* = 0.096
Qatari	51.2	43.7	43.6
Non-Qatari	48.8	56.3	56.4

^**^ *p* < 0.01, ^***^ *p* < 0.001

## 4. Discussion

It has been established that childhood obesity is increasing worldwide [37], with recent trends suggesting that the sharpest upsurges in prevalence are in the GCC countries [9]. It is increasingly accepted that an accumulation of environmental, physiological, personal and lifestyle factors throughout life play a major role in CVD morbidity and mortality [38]. The growing numbers of obese children and adolescents worldwide demand an investment in primary and secondary prevention of obesity and being overweight in youth [39]. However, to intervene effectively, it is necessary to characterize and understand the nature, strength and interactions between biological, psychological, environmental, lifestyle and CVD risk factors in youth.

This study aimed at exploring the prevalence and clustering of lifestyle (e.g., physical activity and sedentary behaviours) and health habits (e.g., healthy and unhealthy dietary habits) of young adults at Qatar University. The study also aimed at identifying demographic characteristics and differences within the clusters, whilst also exploring the reasons for participation in PA, as well as barriers for non-participation. The findings from this study provide substantive information on PA, sedentary behaviour and health habits among students. The current study is the first of its kind to be carried out on this segment of the population (*i.e*., Qatar University students).

The results from the current study appear to indicate that there are greater percentages of university males and females who are classified as overweight/obese compared to previous studies. In the current study, 39.5% of males and 38.5% of females were found to be overweight/obese compared to 31.4% of males and 23.9% of females in the study by Bener and Kamal [40] on youth aged 6–18 years. The percentages of overweight/obese young adults in the present investigation are higher than those reported by other authors on younger samples of the population. For example, Rizk and Yousef’s [41] cross-sectional study on Qatari primary school students (aged 6–11 years) found that 31.7% of boys and 32.8% of girls were overweight/obese. Kerkadi *et al*. [42] reported that the prevalence of being overweight/obese was 44.7% for Qatari boys and 43.3% for girls aged 11 years. On the other hand, Davallow *et al*., [43] reported that the overall prevalence of obesity among Grade 4, 8 and 11 youth in independent schools in Qatar was 23.3%, 22.4% and 17.5%, respectively. The figures clearly demonstrate that the percentage of youth classified as overweight/obese is lower than that found in this investigation among university students. Within the university population, there is even more evidence in this study that obesity is increasing, as the percentage of overweight/obese female students in Year 1 was 32% and by Year 4, this had risen to 46%. Similarly, the percentage of overweight/obese male students was 36% in Year 1, and by Year 4 this figure had risen to 55%.

The results of this study indicate that concomitant with the rising obesity levels reported here, there is an increase in sedentary behaviour amongst university students as they progress through the four years of the university study (Table 2). The percentage of students who are physically active, for example, drops from 42.1% in Year 1 to 20.8% in Year 4, a trend that suggests a worsening problem amongst university students over their time at university. These values are cause for concern with regard to students’ health as they progress from Year 1 to Year 4. Additionally, males spent 4 h and females 4 h 26 min per day engaged in TV viewing and computer use. This is much higher than the 3 h reported by Bener *et al*., [10] in their study on students aged six to 18 years. A significant positive association was evident between BMI and computer-use/sedentary behaviour. This appears to be in line with the results reported by Al-Nakeeb *et al*., [12] on youth in Saudi Arabia.

With regard to the reasons why students participate in physical activity, the results revealed that health (62.2% males and 45.9% females, respectively) followed by losing weight (17.4% and 28.5%, respectively) were the main reasons. Moreover, students reported that the major barrier for non-participation in PA or sport was lack of time (70.3% males and 61.3% of females, respectively). However, it was evident that the reasons cited for non-participation cannot be justified due to the fact that students appear to spend a significant proportion of the day engaged in sedentary activities, including TV viewing and computer use. Another reason for non-participation in PA included “health reasons”, which again presents a worrying picture regarding university students’ lifestyles and the future vitality of the study population.

The cluster analyses revealed that there were three distinct clusters in this study based on the variables being investigated: Cluster 1 “high risk factors” consisted mainly of female students (70.6%) with a mean age of 20.4 years. This cluster also comprised 42.2% of the entire study population. This cluster was shaped by poor engagement with healthy dietary practices, the highest consumption of unhealthy foods, low PA levels and the highest BMI. Sedentary behaviour, such as TV viewing (3 h 16 min) was also greatest in this cluster compared to the other two clusters. Cluster 2, “moderate risk factors”, consisted mainly of male students (69%) with a mean age of 20.9 years. This cluster comprised 24.4% of the study population and was shaped by moderate intake of fruit and vegetable, breakfast consumption (three-times per week) and moderate consumption of milk. This group had moderate intake of sugary food, fast foods, fried potatoes and an average consumption of two energy drinks per week. This cluster, however, did report engagement in the most PA per week, had the lowest TV and computer use and the lowest BMI. Cluster 3 “low risk factors” consisted mainly of female students (58.7%) with a higher mean age of 22.4 years. This cluster comprised 33.3% of the entire study population. This cluster was shaped by the highest engagement in healthy dietary practices, the lowest engagement with unhealthy dietary practices, moderate levels of PA per week and a mean BMI of 24.9 (Table 4). This BMI is greater than that in Cluster 2, but lower than the BMI in Cluster 1. Similarly, TV viewing was greater than in Cluster 2, but less than in Cluster 1. Computer viewing, specifically, was greater in this cluster compared to Clusters 1 and 2.

## 5. Conclusions

The findings from this study reaffirm the notion that health practices tend to occur in clusters rather than in isolation. One should then consider the patterns within these clusters of behaviours when planning policies and designing intervention strategies regarding lifestyle, nutritional habits, PA and obesity in young adults. The need for action in the form of early screening and effective intervention among young adults in Qatar is urgent [42]. These interventions need to follow robust protocols, such as the multi-disciplinary Intervention Mapping (IM) Protocol [44] utilized in the Dutch Obesity Intervention in Teenagers (DoiT). The current research provides valuable data that could be used by policy makers, university management and faculty alike to address issues concerning the health and wellbeing of students, such as limiting the progression of CVD diseases and improving the health of future generations in Qatar. This could be achieved through intervention strategies that lead to changing the built environment and affecting behavioural modification of student’s lifestyle and health habits.
